# Positive selection in the hemagglutinin-neuraminidase gene of Newcastle disease virus and its effect on vaccine efficacy

**DOI:** 10.1186/1743-422X-8-150

**Published:** 2011-03-31

**Authors:** Min Gu, Wujie Liu, Lijun Xu, Yongzhong Cao, Chunfeng Yao, Shunlin Hu, Xiufan Liu

**Affiliations:** 1 Animal Infectious Disease Laboratory, School of Veterinary Medicine, Yangzhou University, Yangzhou 225009, PR China

## Abstract

**Background:**

To investigate the relationship between the selective pressure and the sequence variation of the hemagglutinin-neuraminidase (HN) protein, we performed the positive selection analysis by estimating the ratio of non-synonymous to synonymous substitutions with 132 complete HN gene sequences of Newcastle disease viruses (NDVs) isolated in China.

**Results:**

The PAML software applying a maximum likelihood method was used for the analysis and three sites (residues 266, 347 and 540) in the HN protein were identified as being under positive selection. Codon 347 was located exactly in a recognized antigenic determinant (residues 345-353) and codon 266 in a predicted linear B-cell epitope. Substitutions at codon 540 contributed to the N-linked glycosylation potential of residue 538. To further evaluate the effect of positively selected sites on the vaccine efficacy, we constructed two recombinant fowlpox viruses rFPV-JS6HN and rFPV-LaSHN, expressing the HN proteins from a genotype VII field isolate Go/JS6/05 (with A266, K347 and A540) and vaccine strain La Sota (with V266, E347 and T540), respectively. Two groups of SPF chickens, 18 each, were vaccinated with the two recombinant fowlpox viruses and challenged by Go/JS6/05 at 3 weeks post-immunization. The results showed that rFPV-JS6HN could elicit more effective immunity against the prevalent virus infection than rFPV-LaSHN in terms of reducing virus shedding.

**Conclusions:**

The analysis of positively selected codons and their effect on the vaccine efficacy indicated that the selective pressure on the HN protein can induce antigenic variation, and new vaccine to control the current ND epidemics should be developed.

## Background

Newcastle disease (ND) is notorious for its devastations to the world poultry industry and listed as one of the notifiable terrestrial animal diseases by the World Organization for Animal Health (Office International des Epizooties). The causative agent, Newcastle disease virus (NDV), also known as avian paramyxovirus serotype 1, is a member of the family Paramyxoviridae [1]. The virus genome is a non-segmented, single-strand, negative sense RNA which codes for six major proteins including nucleocapsid protein (NP), phosphoprotein (P), matrix protein (M), fusion protein (F), hemagglutinin-neuraminidase (HN), and large RNA-directed RNA polymerase (L), in the order from the 3' to 5' terminus [2]. Since its emergence in fowls in 1926, NDV has undergone substantial genetic evolution and has developed into several distinct genotypes (I to IX) [3,4]. Among these, genotype VII is considered to be responsible for the severe outbreaks in Western Europe [5], South Africa and Southern Europe [3], and East Asia [6,7] in the 1990s. Presently, the genotype VII NDV is still prevalent in China [4,8-10].

Although the cleavability of F protein is pivotal to NDV pathogenicity [11,12], recent studies have shown that HN protein also contributes to tissue tropism and virulence [13]. HN is an important immunoprotective glycoprotein on the envelope of ND virions and responsible for essential viral functions, such as binding to sialic acid-containing cell receptors, facilitating the fusion activity of the F protein and removing sialic acid to release progeny virus particles [14]. Despite the critical role that HN protein plays in NDV immunity and pathogenesis, the positive selection pressure acting on HN during the viral evolution has not been well analyzed.

The ratio of non-synonymous (*d*_*N*_) to synonymous (*d*_*S*_) substitutions (ω = *d*_N_/*d*_S_) provides an important means for studying the selective pressure at the protein level, with ω = 1 denoting neutral mutations, ω < 1 purifying selection, and ω > 1 diversifying positive selection. As a high proportion of amino acids in many proteins is often largely invariable (with ω close to 0) due to strong structural and functional constraints, approaches conferring an average ω over all codons across the gene are not sensitive enough to detect positive selection [15]. The program PAML [16,17], which applies a maximum likelihood (ML) criterion and a few simple models allowing for heterogeneous ω ratios among sites, has been considered an efficient integrated method to estimate positive selection and has been commonly used to study virus evolution [18-21]. In this paper, the selective pressure on NDV HN protein was examined using 132 complete HN sequences (Chinese isolates), including 106 retrieved from GenBank (up to 14 April, 2009) and the other 26 obtained from field isolates. Based on the analysis, three codons of HN were identified under positive selection and their potential effect on the routine vaccine efficacy was then evaluated.

## Materials and methods

### Viruses

Four pigeon isolates: NDV03-018, NDV03-044, NDV05-028 and NDV05-029 [22], were kindly provided by Dr. Zhiliang Wang (China Animal Health and Epidemiology Center). Two chicken isolates, QH-1/79 and QH-4/85 [23], were obtained from Dr. Dianjun Cao (Harbin Veterinary Research Institute, Chinese Academy of Agricultural Sciences). Twenty field strains were isolated from diseased chicken and goose flocks in China during 2005-2006. All of these viruses were subjected to three rounds of plaque-purification in chick embryo fibroblast (CEF) monolayers and subsequently propagated in 10-day-old specific pathogen free (SPF) chicken embryos. Infective allantoic fluid containing virus stocks was aliquoted and stored at -80°C before use.

### RNA preparation, PCR, and sequencing

Viral RNAs were extracted directly from the allantoic fluid with the Trizol LS reagent (Invitrogen, Carlsbad, CA), following the manufacturer's instructions. Reverse transcription (RT) was conducted with random primers, and PCR was performed with a pair of primers (sense: 5'-CTTCACAACATCCGTTCTACC-3', antisense: 5'-ACCTTCCGAGTTTTATCATTCT-3') to amplify the full-length HN gene of NDV. The PCR products were purified with a DNA purification kit (QIAGEN, Hilden, Germany) and sequenced directly using the ABI PRISM BigDye Terminator v3.1 Cycle Sequencing kit (Applied Biosystems, Foster City, CA).

### Sequence information and phylogenetic analysis

GenBank accession numbers assigned to the 26 strains characterized in the present study were as follows: FJ751918, FJ751919, FJ766528, EF666110, GQ338309-GQ338311, and EU044809-EU044827. In addition, the other 106 full-length HN sequences of NDV isolates from China (vaccine strains of La Sota and Mukteswar were included as they are used extensively in poultry flocks, while recombinant strains were excluded to ensure the accuracy of detecting positive selection at amino acid sites [24]) were retrieved directly from GenBank and their accession numbers were listed in Table 1. All the 132 HN sequences were edited and aligned with the Lasergene software (DNASTAR Inc., Madison, WI). The GTR (general time reversible) + I (invariable sites) + G (gamma distribution) evolutionary model was selected as the optimal nucleotide substitution model with the program Modeltest 3.7 [25]. Phylogenetic tree was then constructed by employing the ML method implemented in PAUP* version 4.0b [26] and neighbor-joining (NJ) method in MEGA version 4.0 [27]. The robustness of the statistical support for the tree branch was evaluated by 1000 bootstrap replicates. The online server, BepiPred 1.0 [28], was used to predict the position of linear B-cell epitopes of all the HN sequences.

**Table 1:** Accession numbers of the 106 complete HN gene sequences of NDVs isolated in China that were directly retrieved from GenBank (* sequences with lysine (K) at residue 347)

Accession Number	Accession Number	Accession Number	Accession Number	Accession Number
DQ060053	EU346660	FJ004152	DQ314571-DQ314572	DQ368684*
DQ228924	EU546165	FJ240169	DQ485229-DQ485231	DQ485270*
DQ228931	AF204872	AF456429-AF456434	DQ485262-DQ485268	DQ682452*
DQ485272	AY135171	EF211815-EF211818	DQ469832-DQ469833	DQ228925-DQ228930*
DQ486859	AY253912	FJ011444-FJ011447	DQ234586-DQ234587	DQ228932-DQ228935*
DQ659677	AY351959	FJ386392-FJ386396	DQ682447-DQ682451	DQ234584-DQ234585*
DQ858355	AY997298	DQ023148-DQ023155	DQ023156*	DQ234590-DQ234592*
EF201805	EF540730	DQ023554-DQ023557	DQ023558*	DQ469830-DQ469831*
EU649675	EF175144	DQ023559-DQ023560	DQ234579*	FJ217668-FJ217669*
EU481973	EF141104	DQ234580-DQ234583	DQ234588*	

### Positive selection detection

To estimate the selective constraints on the HN protein, the codeml program of the PAML package (version 4) [16] was utilized to calculate the site-to-site variation in ω. Two nested site-specific models, consisting of a neutral model that does not allow positive selection (ω≤1) and an alternative model that permits positive selection (ω > 1), were compared. As recommended [15], the following models were used: M0 (one-ratio) v. M3 (discrete) and M7 (beta) v. M8 (beta & ω). M0 assumes a constant ω for all codons whereas M3 allows for discrete classes of sites with different ω ratios. M7 supposes a beta distribution with 10 categories of ω over sites, each corresponding to a unique ω value that is always less than 1 while M8 has an extra category with ω > 1. Then the log likelihood values for each pair of the above nested models were compared by a likelihood ratio test (LRT) [15,17], in order to assess whether the model allowing for positive selection is significantly more suitable for the data. Finally, the Bayes empirical Bayes (BEB) procedure [29] was used to infer the particular codons under positive selection and to calculate their posterior probabilities.

### Animal experiment

To further investigate the effect of positively selected sites on vaccine efficacy against the prevalent NDVs, Go/JS6/05 (field NDV strain) and La Sota (the most widely used vaccine strain in China) were chosen to construct corresponding recombinant fowlpox viruses (rFPVs) expressing each HN gene based on the transfer vector pP12LS developed by Sun et al [30]. The expression was identified by indirect immunofluorescence assay (IFA) in secondary CEF cultures using anti-NDV polyclonal antibody as previously described [31], and the levels of HN expression were further compared between the two generated rFPVs by flow cytometry [32] on DF-1 cells (a stable cell line of CEF) at a multiplicity of infection (MOI) of 5. Subsequently, two groups of five-day-old SPF White Leghorn chickens (18 birds/group, Beijing Merial Vital Laboratory Animal Technology, Beijing, China) were immunized respectively with the above two rFPVs at a dose of 1 × 10^4 ^PFU. A third group was served as a mock-vaccinated control. Three weeks later, all chickens were challenged oculonasally with 100 μL of PBS-diluted allantoic fluid containing 1 × 10^5 ^EID_50 _of Go/JS6/05. Tracheal and cloacal swabs were collected on days 3, 5 and 7 post-challenge (p.c.). Furthermore, six chickens from each vaccinated group were sacrificed humanely on day 5 p.c., and tissue samples including liver, brain, spleen, kidney, trachea and lung were collected. The swabs were immersed in PBS with antibiotics (8000 U/mL ampicilin, 5 mg/mL streptomycin and kanamycin, pH 7.2), and stored at -80°C until analyzed. The recovery of the challenged virus in these swabs or organ samples was confirmed by inoculation into embryonated chicken eggs. All animal work was approved by the Jiangsu Administrative Committee for Laboratory Animals (Permission number: SYXK-SU-2007-0005).

## Results

### Sequence analysis

**Figure 1:**
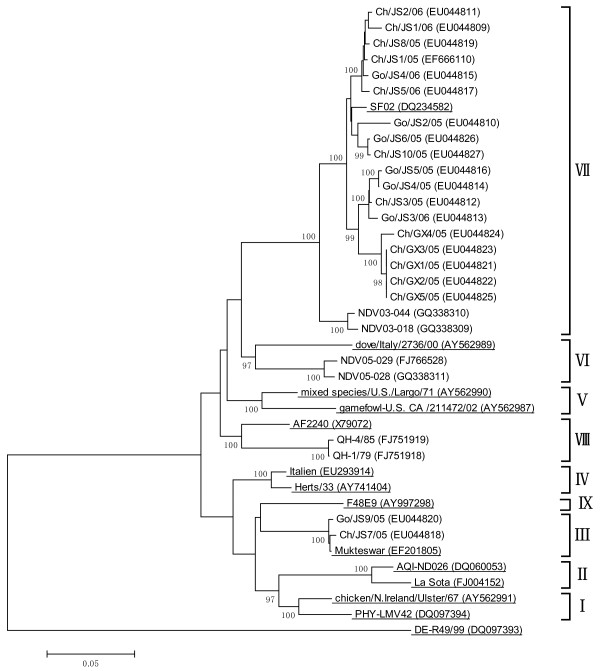
**Phylogenetic tree of the 26 NDV field isolates based on the full coding region of the HN gene**. Reference sequences representing recognized genotypes were denoted with their taxon names underlined. The tree was generated using the neighbor-joining method implemented in program MEGA 4.0. The Class I NDV strain DE-R49/99 was served as an outgroup. The scale indicates the branch length based on the number of nucleotide substitutions per site and the numbers at nodes indicate bootstrap values calculated by resampling of 1000 replicates.

The full coding region of the 132 HN genes analyzed in this study exhibited diverse phylogenetic phenotypes in Chinese poultry flocks, containing six of the nine recognized genotypes (II, III, VI, VII, VIII and IX), with the overwhelming majority (113/132) belonged to genotype VII (Additional file 1, Table S1). In addition, the phylogenetic tree (Figure 1) showed that our 26 sequences belonged to four different genotypes as follows: III (Ch/JS7/05 and Go/JS9/05), VI (NDV05-028 and NDV05-029), VIII (QH-1/79 and QH-4/85) and the remaining 20 fell into genotype VII.

### Detection of positive selection

An ML method implemented in the software package PAML was used for the identification of any positive selection on the HN protein of NDV. The log likelihood differences between M0 and M3, as well as M7 and M8 were found to be significant. Models that permitted positive selection showed a better fit to the data and contained a class of codons with the non-synonymous to synonymous substitutions ratio greater than one (ω > 1), indicating the existence of positive selection (Table 2). A further analysis on the amino acids most likely responsible for the detected non-neutral pattern revealed that three codons, 266, 347 and 540, were under positive selection identified by both M3 and M8, with the posterior probability over 95% for residue 266 and 347, and over 90% for residue 540. As the M0-M3 comparison is more a test of heterogeneity in the ω value among sites and not actually a test of positive selection [33], only the results obtained by M8 were investigated further.

**Table 2:** Parameter estimates and likelihood values corresponding to the results from different models

Model	lnL	dN/dS	Estimated parameters	**Df **^**a**^	**2△l **^**b**^	**Positively selected codons **^**c**^
M0 (one-ratio)	-13384.287	0. 225	ω = 0. 225	4		None
M3 (discrete)	-13246. 011	0. 228	p_0_= 0.656, p_1_= 0.310, p_2_= 0.034 ω_0_= 0.069, ω_1_= 0.446, ω_2 _= 1.313^d^		276.552 (p < 0.01)	**266**, **347**, 540
M7 (beta)	-13253. 972	0. 227	p = 0.486, q = 1.635	2		Not allowed
M8 (beta& ω)	-13247. 102	0. 228	p_0_= 0.968, p_1_= 0.032 p = 0.662, q = 2.730, ω = 1.312		13.740 (p < 0.01)	**266**, **347**, 540

^a ^Df: degree of freedom.

^b ^2△l: Log likelihood difference between nested models using the χ2-test.

^c ^Codons estimated to be under positive selection at the 90% level. Numbers in bold denote codons with posterior probabilities over 95%.

^d ^Underlined parameters are evidence of positive selection.

### Amino acid variations of positively selected codons

As shown in Table 3, each of the three positively selected codons identified by M8 exhibited diversity in amino acid substitutions which could induce the variations in hydrophobicity or charge. In particular, codon 266 contained the most abundant amino acid substitutions. According to program BepiPred 1.0, a fragment of the HN sequence covering residues 255 to 265, sometimes extending to residue 267 or even 270, was detected as a potential B-cell epitope. And codon 266 was involved in the above predicted epitope of 119 HN sequences, including all the 113 genotype VII viruses (Additional file 1, Table S1).

**Table 3:** Amino acid variations of the positively selected codons identified by M8

Codon	Residue	**Substitution frequency **^**a**^	**Hydrophobicity **^**b**^	Charge	**site ω( ± S.E.)**^**c**^
266	A	91	Y	Neutral	1. 477 ± 0. 125
	T	23	N	Neutral	
	V	10	Y	Neutral	
	I	4	Y	Neutral	
	P	2	Y	Neutral	
	D	1	N	Negative	
	S	1	N	Neutral	
347	E	62	N	Negative	1. 485 ± 0. 100
	G	41	Y	Neutral	
	K	28	N	Positive	
	N	1	N	Neutral	
540	A	94	Y	Neutral	1. 438 ± 0. 202
	V	23	Y	Neutral	
	T	15	N	Neutral	

^a ^Numbers represent the occurrence times of each specific residue among the 132 HN sequences.

^b ^Y stands for hydrophobic and N for hydrophilic.

^c ^Estimated ω value at single site with standard error.

### Generation of rFPVs expressing HN genes

**Figure 2:**
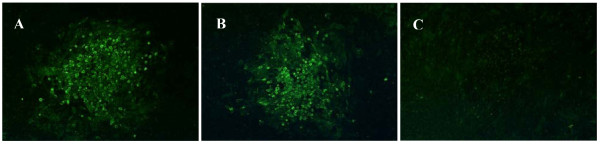
**Identification of HN protein expressed in the recombinant fowlpox virus (rFPV) by indirect immunofluoresence assay (IFA)**. Chick embryo fibroblast (CEF) cells were infected with rFPV-LaSHN (A), rFPV-JS6HN (B), or wild-type parental FPV (C) at a multiplicity of infection (MOI) of 0.01. Sixty hours post-infection, IFA was performed using polyclonal anti-NDV chicken serum and FITC-conjugated rabbit anti-chicken IgG as primary and secondary antibody, respectively. The fluorescence was observed under an inverted fluorescence microscope.

Two rFPVs, rFPV-LaSHN and rFPV-JS6HN respectively expressing the HN proteins of vaccine strain La Sota (with V266, E347 and T540) and genotype VII field isolate JS-06/05 (with A266, K347 and A540) were generated by homologous recombination between the corresponding transfer plasmid and the wild-type parental FPV. Fluorescence was readily observed in CEF cells transfected with either rFPV-LaSHN (Figure 2A) or rFPV-JS6HN (Figure 2B), demonstrating the expression of the inserted HN gene. The flow cytometric analysis indicated that there was no significant difference in the expression levels of HN between rFPV-LaSHN and rFPV-JS6HN on DF-1 cells at 24 hours post-infection (data not shown).

### Protective efficacies of rFPV-JS6HN and rFPV-LaSHN

On day 21 after immunization, effective antibody responses were induced in both rFPV vaccinated groups, with higher hemagglutinin-inhibition (HI) titers to the homologous antigen, and serum from birds inoculated with rFPV-LaSHN reacted poorly with Go/JS6/05 (Table 4). On day 5 p.c., virus replication in different visceral organs of rFPV-JS6HN or rFPV-LaSHN vaccinated chickens was examined by inoculation into 10-day-old embryonated chicken eggs. As shown in Table 4, the frequencies of virus isolation in the brain, spleen and lung from rFPV-LaSHN group were higher than that of the rFPV-JS6HN, though no statistically significant difference was observed.

**Table 4:** HI titers to NDV at day pre-challenge and virus detection in visceral organs from different rFPV immunized groups on day 5 p.c.

Vaccine group	**HI titer ± standard deviation **^**a**^		**Virus detection (positive/total) **^**c**^					
		
	Go/JS6/05	La Sota	Liver	Brain	Spleen	Kidney	Trachea	Lung
rFPV-JS6HN	**3.80 ± 0.92 **^**b**^	3.30 ± 1.16	0/6	3/6	3/6	4/6	0/6	2/6
rFPV-LaSHN	1.50 ± 0.85	**3.90 ± 0.57**	0/6	5/6	6/6	4/6	0/6	5/6

^a ^HI titers to Go/JS6/05 or La Sota were evaluated on day 21 post vaccination.

^b ^titers to homologous antigens were noted in bold.

^c ^Virus replication was detected on day 5 post challenge by inoculation into 10-day-old SPF embryonated chicken eggs.

Each of the rFPV-JS6HN and rFPV-LaSHN vaccinated chickens was fully protected against mortality after challenge, whereas unvaccinated birds died within 5 days p.c.. On day 3 p.c., virus shedding from the cloaca and trachea showed that both the rFPV vaccines remarkably decreased the level of virus excretion from cloaca, and that the rFPV-JS6HN group could significantly reduce the virus recovery rates from trachea when compared with the rFPV-LaSHN group. Moreover, on days 5 and 7 p.c., the frequencies of virus isolation from cloaca in rFPV-JS6HN vaccinated birds were much lower than that in rFPV-LaSHN immunized fowls (Table 5).

**Table 5:** Virus recovery of swab samples from different rFPV immunized groups challenged with NDV Go/JS6/05

Vaccine group	No. of birds shedding/total no. of birds on day after challenge:						Mortality
		
	3		5		7		
				
	Trachea	Cloaca	Trachea	Cloaca	Trachea	Cloaca	
rFPV-JS6HN	11/18^B^	4/18^B^	0/18^A^	1/18^A^	0/12^A^	0/12^A^	0/18^A^
rFPV-LaSHN	16/18^AB^	7/18^B^	1/18^A^	9/18^B^	0/12^A^	2/12^A^	0/18^A^
Unvaccinated	17/18^A^	14/18^A^	NS	NS	NS	NS	18/18^B^

^A, B ^Different superscript letters denote significant difference between treatment groups within the column; Fisher's exact test, *P *< 0.05.

NS: No survivors.

## Discussion

Positive selection is an evolutionary process that could drive the fixation of emerging advantageous mutations in the population with higher frequencies compared to the wild-type allele [34]. Therefore, identifying proteins or protein domains that experiencing adaptive selection will improve the understanding of their genomic functions and the recognition of genetic variation that leads to phenotypic diversity [35].

Observations from previous genetic and antigenic studies of viruses such as FMDV (foot-and-mouth disease virus) [18], HIV-1 (human immunodeficiency virus type 1) [19], RHDV (rabbit hemorrhagic disease virus) [20] and influenza B virus [21], have indicated that signatures of positive selection are generally functionally important and/or associated with antigenicity. To date, seven antigenic determinants that form a continuum on HN protein have been characterized by a panel of monoclonal antibodies (mAbs) against the HN of the velogenic Australia-Victoria/32 (AV) strain, including the amino acids positions 193, 194, 201, 263, 287, 321, 332, 333, 345, 347, 350, 353, 356, 494, 513, 514, 516, 521 and 569 [36,37]. One of our positively selected sites, codon 347, was located exactly in those defined epitopes. In the present study, both La Sota and Mukteswar, which are widely used vaccine strains in China, have a glutamic acid (E) occupied codon 347, in contrast to that with a lysine (K) substitution resulting in the opposite residual charge exclusively in genotype VII viruses (accession numbers in italic in Table 1). Furthermore, as referred to recent work of Cho et al [38] and Hu et al (2009) [39], it is reasonable to postulate that the emergence of E347K substitution might be closely related to the host immune pressure. However, the codon 266 was not included in the aforementioned antigenic sites [36,37], instead, it was involved in a predicted linear B-cell epitope that simultaneously held the epitope residue 263, suggesting that site 266 may lie in some antigenic regions yet to be recognized.

N-linked glycosylation, one of the most common forms of protein post-translational modifications, is known to be correlated with viral infectivity and immune escape [40]. There are six potential N-glycosylation sites (amino acids 119, 341, 433, 481, 508 and 538) in the HN protein of the AV strain [41]. In our analysis, positive selection was detected at codon 540, which comprised three different amino acids: alanine (A), valine (V) and threonine (T). Residue 538 was conserved with asparagine (N) in all the 132 HN sequences and tended to be a putative N-glycosylation site if T was present at site 540. However, the vast majority of prevalent strains owned A or V at codon 540 (Additional file 1, Table S1), which would deprive the possibility of N538 being glycosylated. The exact function of the resulted deglycosylation at site 540 remains unknown and needs to be further explored.

Compared to vaccine strain La Sota, most genotype VII NDV isolates possessed different amino acids at the three identified positively selected sites. To further evaluate the effect of those sites on the vaccine protective efficacy, Go/JS6/05 was chosen together with La Sota for the recombinant fowlpox-virus construction. Before challenge, serum collected from the rFPV-LaSHN immunized chickens displayed lower HI titers to Go/JS6/05 than to La Sota (Table 4), which may suggest that substitutions at the positively selected sites are partially responsible for the antigenic variation between the two HN proteins. After challenge, virus shedding results showed that the rFPV-JS6HN could prevent the excretion of the challenged virus more efficiently than rFPV-LaSHN, indicating that the positively selected sites on the HN protein could affect the vaccine immune efficacy against the prevalent NDV infection.

Although an intensive vaccination program against ND has been executed in China in the last few decades, epidemic infections with velogenic genotype VII NDV in vaccinated birds are still frequently reported in recent years [4,8-10]. Currently, the most extensively used vaccine strains, such as La Sota (genotype II) and Mukteswar (genotype III), were isolated and characterized in the 1940s and belonged to the "early" genotypes (I-IV and IX), which have evident amino acid sequence divergence from the "late" ones (V-VII), especially genotype VII [42]. The results in this study suggest that positive selection may play a role in the formation of such differentiation and even induce antigenic variations compared with the vaccine strains. Therefore, new vaccine to better control the ND epizootics of prevalent NDV strains carrying novel variations at identified positively selected sites should be developed to meet the challenge.

## Competing interests

The authors declare that they have no competing interests.

## Authors' contributions

MG carried out the study design, phylogenetic analysis and positive selection, and drafted the manuscript. WL and LX performed the construction of recombinant fowlpox viruses and participated in the whole procedure of the animal experiment. YC guided the analysis of positively selected sites. CY participated in sample collection and virus isolation from diseased poultry flocks, and HN gene sequencing. SH contributed to the design of the study and revision of the manuscript. XL conceived of the study, provided consultation and coordination, and helped to draft the manuscript. All authors read and approved the final manuscript.

## Supplementary Material

Additional file 1 Table S1: Background information of the 132 HN sequences investigated in the study Click here for file
