# Supplementary material for: Positive selection in the hemagglutinin-neuraminidase gene of Newcastle disease virus and its effect on vaccine efficacy
Source: Virol J. 2011 Mar 31;8:150. doi: 10.1186/1743-422X-8-150 (PMC3101160; doi:10.1186/1743-422X-8-150)
Supplement: Additional file 1 — Table S1: Background information of the 132 HN sequences investigated in the study [file 1743-422X-8-150-S1.DOC]

**Additional file**

**Table S1. Background information of the 132 HN sequences investigated in the study**

| GenBank  Accession Number | | Genotype | Amino Acid  substitutions | | | Potential linear B-cell epitope  around residue 266 |
| --- | --- | --- | --- | --- | --- | --- |
|  |  |  | 266 | 347 | 540 |  |
|  | *EU044809 (Ch/JS1/06)* | Ⅶ | T | G | A | 255-270 |
|  | *EU044810 (Go/JS2/05)* | Ⅶ | A | K | A | 254-270 |
|  | *EU044811 (Go/JS2/06)* | Ⅶ | A | G | A | 255-270 |
|  | *EU044812 (Ch/JS3/05)* | Ⅶ | A | E | A | 255-267 |
|  | *EU044813 (Go/JS3/06)* | Ⅶ | A | E | A | 255-272 |
|  | *EU044814 (Go/JS4/05)* | Ⅶ | A | E | A | 255-267 |
|  | *EU044815 (Go/JS4/06)* | Ⅶ | A | G | A | 255-270 |
|  | *EU044816 (Go/JS5/05)* | Ⅶ | A | E | A | 255-267 |
|  | *EU044817 (Ch/JS5/06)* | Ⅶ | A | G | A | 255-270 |
|  | *EU044819 (Ch/JS8/05)* | Ⅶ | A | G | A | 255-270 |
|  | *EU044821 (Ch/GX1/05)* | Ⅶ | A | G | A | 255-267 |
|  | *EU044822 (Ch/GX2/05)* | Ⅶ | A | G | A | 255-267 |
|  | *EU044823 (Ch/GX3/05)* | Ⅶ | A | G | A | 255-267 |
|  | *EU044824 (Ch/GX4/05)* | Ⅶ | A | G | A | 255-267 |
|  | *EU044825 (Ch/GX5/05)* | Ⅶ | A | G | A | 255-267 |
|  | *EU044826 (Go/JS6/05)* | Ⅶ | A | K | A | 255-267 |
|  | *EU044827 (Ch/JS10/05)* | Ⅶ | A | N | A | 255-267 |
|  | *EF666110 (Ch/JS1/05)* | Ⅶ | A | G | A | 255-270 |
|  | *GQ338309 (NDV03-018)* | Ⅶ | T | G | A | 255-270 |
|  | *GQ338310 (NDV03-044)* | Ⅶ | T | E | A | 255-270 |
|  | *FJ766528 (NDV05-029)* | Ⅵ | P | G | V | 255-265 |
|  | *GQ338311 (NDV05-028)* | Ⅵ | S | G | V | 255-265 |
|  | *FJ751918 (QH-1/79)* | Ⅷ | T | E | V | 255-270 |
|  | *FJ751919 (QH-4/85)* | Ⅷ | T | E | V | 256-270 |
|  | *EU044820 (Go/JS9/05)* | Ⅲ | V | E | T | 255-265 |
|  | *EU044818 (Ch/JS7/05)* | Ⅲ | I | E | T | 255-265 |
|  | DQ314571 | Ⅶ | A | E | V | 255-267 |
|  | DQ314572 | Ⅶ | A | E | V | 255-267 |
|  | DQ023153 | Ⅶ | A | E | A | 254-267 |
|  | DQ023155 | Ⅶ | A | E | A | 255-267 |
|  | DQ023154 | Ⅶ | A | E | A | 255-267 |
|  | EU481973 | Ⅶ | A | E | A | 255-267 |
|  | DQ228928 | Ⅶ | A | K | A | 255-270 |
|  | DQ023156 | Ⅶ | A | K | A | 255-270 |
|  | AY253912 | Ⅶ | A | E | A | 255-267 |
|  | DQ228924 | Ⅶ | A | G | A | 255-270 |
|  | EF211815 | Ⅶ | A | G | A | 255-270 |
|  | DQ469832 | Ⅶ | A | G | A | 255-270 |
|  | EU649675 | Ⅶ | A | G | A | 255-270 |
|  | EF211817 | Ⅶ | A | G | A | 255-270 |
|  | DQ469833 | Ⅶ | A | G | A | 255-270 |
|  | FJ011446 | Ⅶ | A | G | A | 255-270 |
|  | FJ011445 | Ⅶ | A | G | A | 255-270 |
|  | FJ011444 | Ⅶ | A | G | A | 255-270 |
| GenBank  Accession Number | | Genotype | Amino Acid  substitutions | | | Potential linear B-cell epitope  around residue 266 |
|  |  |  | 266 | 347 | 540 |  |
|  | FJ011447 | Ⅶ | A | G | A | 255-270 |
|  | FJ240169 | Ⅶ | A | G | A | 255-270 |
|  | EF211816 | Ⅶ | A | G | A | 255-270 |
|  | DQ682450 | Ⅶ | A | E | A | 255-270 |
|  | DQ682449 | Ⅶ | A | E | A | 255-270 |
|  | DQ682448 | Ⅶ | A | E | A | 255-270 |
|  | DQ234581 | Ⅶ | A | E | A | 255-271 |
|  | DQ234583 | Ⅶ | A | E | A | 255-271 |
|  | DQ234580 | Ⅶ | A | E | A | 255-267 |
|  | DQ228925 | Ⅶ | A | K | A | 255-267 |
|  | DQ228929 | Ⅶ | A | K | A | 255-267 |
|  | DQ234584 | Ⅶ | T | K | A | 257-270 |
|  | DQ228926 | Ⅶ | T | K | A | 255-270 |
|  | DQ228934 | Ⅶ | A | K | A | 254-267 |
|  | DQ228933 | Ⅶ | A | K | A | 255-267 |
|  | DQ023558 | Ⅶ | A | K | A | 255-267 |
|  | FJ217669 | Ⅶ | A | K | A | 255-267 |
|  | FJ217668 | Ⅶ | A | K | A | 255-267 |
|  | DQ485270 | Ⅶ | A | K | A | 255-267 |
|  | DQ682452 | Ⅶ | A | K | A | 255-267 |
|  | DQ469831 | Ⅶ | A | K | A | 255-267 |
|  | DQ469830 | Ⅶ | A | K | A | 255-267 |
|  | DQ234582 | Ⅶ | A | E | A | 255-267 |
|  | DQ023554 | Ⅶ | A | E | A | 255-267 |
|  | DQ023559 | Ⅶ | A | E | A | 255-267 |
|  | DQ023149 | Ⅶ | A | E | A | 255-267 |
|  | AF456434 | Ⅶ | A | E | A | 255-267 |
|  | DQ023148 | Ⅶ | A | E | A | 255-267 |
|  | EF175144 | Ⅶ | A | E | A | 255-267 |
|  | AF431744 | Ⅶ | A | E | T | 255-267 |
|  | AF456431 | Ⅶ | A | E | A | 255-267 |
|  | EF540730 | Ⅶ | D | E | A | 255-270 |
|  | AY351959 | Ⅶ | A | E | A | 255-267 |
|  | DQ234590 | Ⅶ | A | K | V | 255-267 |
|  | DQ234585 | Ⅶ | A | K | V | 255-267 |
|  | DQ228932 | Ⅶ | A | K | V | 255-267 |
|  | DQ234588 | Ⅶ | A | K | V | 255-267 |
|  | DQ228930 | Ⅶ | A | K | V | 255-267 |
|  | DQ228935 | Ⅶ | A | K | V | 255-267 |
|  | DQ228927 | Ⅶ | A | K | V | 255-267 |
|  | DQ368684 | Ⅶ | A | K | V | 255-267 |
|  | DQ234591 | Ⅶ | A | K | V | 255-267 |
|  | DQ234592 | Ⅶ | A | K | V | 255-267 |
|  | DQ234579 | Ⅶ | A | K | V | 255-267 |
|  | EU346660 | Ⅶ | A | G | A | 255-267 |
|  | DQ486859 | Ⅶ | A | E | A | 255-267 |
|  | DQ485272 | Ⅶ | A | E | A | 255-270 |
|  | DQ485231 | Ⅶ | A | E | A | 255-270 |
| GenBank  Accession Number | | Genotype | Amino Acid  substitutions | | | Potential linear B-cell epitope  around residue 266 |
|  |  |  | 266 | 347 | 540 |  |
|  | EF211818 | Ⅶ | A | E | A | 255-267 |
|  | DQ023557 | Ⅶ | A | E | A | 255-267 |
|  | DQ682451 | Ⅶ | A | G | A | 256-270 |
|  | DQ682447 | Ⅶ | A | G | A | 256-270 |
|  | AF456429 | Ⅶ | A | G | A | 256-270 |
|  | AF456430 | Ⅶ | A | G | A | 256-270 |
|  | DQ485229 | Ⅶ | A | G | A | 255-267 |
|  | DQ485267 | Ⅶ | A | G | A | 255-267 |
|  | DQ228931 | Ⅶ | T | E | A | 255-270 |
|  | DQ234587 | Ⅶ | T | E | A | 255-270 |
|  | DQ234586 | Ⅶ | T | E | A | 255-270 |
|  | DQ023556 | Ⅶ | T | E | A | 255-270 |
|  | AF204872 | Ⅶ | T | G | A | 255-270 |
|  | DQ023560 | Ⅶ | T | E | A | 255-270 |
|  | DQ023152 | Ⅶ | T | E | A | 256-270 |
|  | DQ023151 | Ⅶ | T | E | A | 256-270 |
|  | DQ485268 | Ⅶ | T | G | V | 256-270 |
|  | DQ485265 | Ⅶ | T | G | V | 256-270 |
|  | DQ485264 | Ⅶ | T | G | V | 256-270 |
|  | AF456433 | Ⅶ | T | E | V | 256-270 |
|  | DQ485263 | Ⅶ | T | G | A | 256-270 |
|  | DQ659677 | Ⅶ | T | E | A | 256-267 |
|  | EF141104 | Ⅶ | T | E | A | 255-267 |
|  | DQ023150 | Ⅶ | A | E | A | 256-270 |
|  | DQ023555 | Ⅶ | T | E | A | 256-270 |
|  | DQ485262 | Ⅶ | A | G | V | 255-267 |
|  | DQ485230 | Ⅶ | A | G | T | 255-267 |
|  | DQ858355 | Ⅵ | P | G | V | 255-265 |
|  | AY135171 | Ⅱ | V | E | T | 255-267 |
|  | EU546165 | Ⅱ | V | E | T | 255-267 |
|  | DQ060053 | Ⅱ | V | E | T | 255-265 |
|  | FJ386396 | Ⅱ | V | E | T | 255-265 |
|  | FJ386395 | Ⅱ | V | E | T | 255-265 |
|  | FJ386394 | Ⅱ | V | E | T | 255-265 |
|  | FJ386392 | Ⅱ | V | E | T | 255-265 |
|  | FJ386393 | Ⅱ | V | E | T | 255-265 |
|  | **FJ004152 (La Sota)** | Ⅱ | V | E | T | 255-267 |
|  | AY997298 | Ⅸ | I | E | T | 255-267 |
|  | DQ485266 | Ⅲ | I | E | A | 255-265 |
|  | **EF201805 (Mukteswar)** | Ⅲ | I | E | T | 255-265 |

*GenBank accession numbers for the 26 field isolates of NDV were shown in italic.
